# Effects of *Abies sibirica* terpenes on cancer- and aging-associated pathways in human cells

**DOI:** 10.18632/oncotarget.13467

**Published:** 2016-11-19

**Authors:** Anna Kudryavtseva, George Krasnov, Anastasiya Lipatova, Boris Alekseev, Faniya Maganova, Mikhail Shaposhnikov, Maria Fedorova, Anastasiya Snezhkina, Alexey Moskalev

**Affiliations:** ^1^ Engelhardt Institute of Molecular Biology, Russian Academy of Sciences, Moscow, 119991, Russia; ^2^ National Medical Research Radiological Center, Ministry of Health of the Russian Federation, Moscow, 125284, Russia; ^3^ Initium-Pharm, LTD, Moscow, 142000, Russia; ^4^ Institute of Biology of Komi Science Center of Ural Branch of RAS, Syktyvkar, 167982, Russia; ^5^ Moscow Institute of Physics and Technology, Dolgoprudny, 141700, Russia

**Keywords:** Abies sibirica, plant extract, terpenoids, anti-aging effects, anti-cancer effects

## Abstract

A large number of terpenoids exhibit potential geroprotector and anti-cancer properties. Here, we studied whole transcriptomic effects of Abisil, the extract of fir (*Abies sibirica*) terpenes, on normal and cancer cell lines. We used early passaged and senescent none-immortalized fibroblasts as cellular aging models. It was revealed that in normal fibroblasts, terpenes induced genes of stress response, apoptosis regulation and tissue regeneration. The restoration of the expression level of some prolongevity genes after fir extract treatment was shown in old cells. In *Caco-2* and *AsPC-1* cancer cell lines, Abisil induced expression of both onco-suppressors (members of GADD45, DUSP, and DDIT gene families), and proto-oncogenes (*c-Myc*, *c-Jun*, *EGR* and others). Thus, the study demonstrates the potential anti-aging and anti-cancer effects of Abisil on senescent and cancer cell lines.

## INTRODUCTION

It is known that certain plant extracts have geroprotector properties. The ability to prolong the life in animal models observed for extracts of hawthorn [1], *Ginkgo biloba* [2], blueberry [3], *Rosa damascena* [4], ginseng [5], cranberry [6], green tea [7], *Nymphaea* root [8], *Alpinia zerumbet* leaf [9], natto [10], *Rhodiola rosea* [11], black rice [12], garlic [13], apple [14], *Stachys lavandulifolia* [15], and others. Anti-cancer agents can act as anti-aging drugs [16, 17]. Specific subjects of interest are terpenoids (isoprenoids), the largest class of naturel products that consist of more than 30 000 individual compounds [18–20]. They are found in animal, fungi and microbial species, but most of terpenoids are of plant origin [21–23]. Plants produce terpenoids both as primary metabolites and as secondary compounds [24]. Most of terpenoids are derived from a five-carbon precursor isopentenyl diphosphate (IPP) in acetate/mevalonate (Ac-MVA) pathway [25]. However, some of ones are produced via recently discovered non-mevalonate (non-MVA) pathway [26, 27]. Terpenoids have different functions; in plants they are involved in basic cellular processes such as cell growth and development, cellular membrane maintenance, stress response, and specialized metabolism [28, 29]. A wide range of terpenoids have exhibited anti-cancer and geroprotector activities and are the candidate compounds for drug discovery [30–32]. For example, extracts from *Rosa damascene*, that is rich in such terpenoid as citronellol, have been shown to increase lifespan of *Drosophila* by protecting against iron toxicity and enhancing flies resistance to oxidative stress [30, 33, 34]. Betulinic acid, a lupane-type triterpene derived from birch tree (*Betula spp.*), have demonstrated the anti-bacterial, antimalarial, and anti-inflammatory properties, activity against the human immunodeficiency virus (HIV), and cytotoxicity towards cancer cells [35–37]. Moreover, both the anti-aging and anti-cancer properties have been observed for such terpenoids as ursolic, maslinic and oleanolic acids [14, 38–41]. Thus, the anti-cancer activity and geroprotector properties of terpenoids appear to be promising for various therapeutic applications.

We studied whole transcriptomic effects of Abisil, the extract of *Abies sibirica* terpenes (10% bornyl acetate), on human cell lines of colon adenocarcinoma (*Caco-2*), pancreas adenocarcinoma (*AsPC-1*) and human none-immortalized fibroblasts of the 6^th^ and 13^th^ passages from the point of view of potential geroprotector and anti-cancer properties.

## RESULTS AND DISCUSSION

Differentially expressed genes that were affected by drug treatment in different cell lines were identified (Supplementary Table S1). Abisil changed the expression of genes involved in the adaptive cellular stress response (by 2 and more times, FDR<0.05) regardless of the number of passages of normal fibroblasts. Among affected genes are heat shock protein 70 (*HSPA1B*, *HSPA1A*), heme oxygenase-1, metallothionein 1X, and dual specificity phosphatase 2 (*DUSP2*).

DUSP2 dephosphorylates MAPKs are involved in cellular proliferation, apoptosis, differentiation, and stress responses [42]. It is known that such geroprotectors and hormetins as curcumin induces expression of an endoplasmic reticulum-anchored enzyme heme oxygenase-1 [43], which is involved in the adaptive response of human fibroblasts to oxidative and chemical stresses [44, 45]. It utilizes the heme in the various proteins and release ferrous iron [46]. In turn metallothioneins (e.g. MT1X) are induced during cellular stress response involved in detoxification of metal ions [47].

There are also genes associated with the immune response, such as *BHLHE40* [48] and *IFIT2* [49], and factors related to cell differentiation, such as FOSB [50] and TRIB1 [51]. In addition, TRIB1 plays role in lipid metabolism [52]. The upregulated gene *Egr-3* is a transactivator of genes in fibroblasts, associated with tissue remodeling and wound healing [53].

In fibroblasts of both the 6^th^ and 13^th^ passages Abisil suppressed the expression of proapoptotic gene *BMF* [54] as well as molecules of cell adhesion: integrins ITGB7, ITGAM [55] and cell surface glycoprotein MUC13 [56].

**Figure 1:**
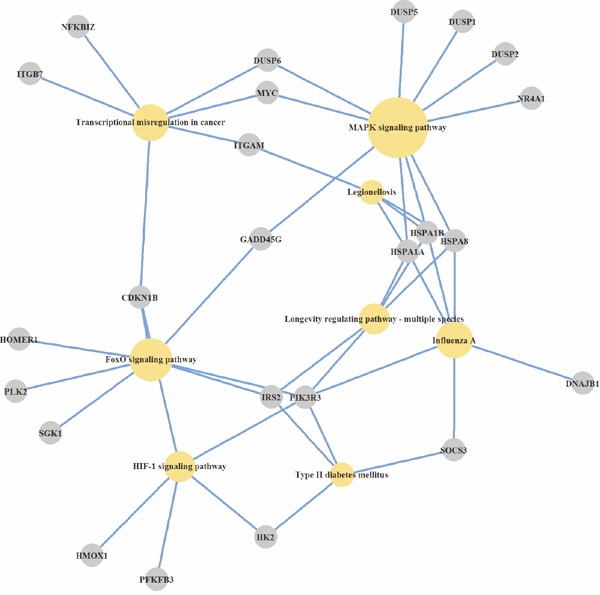
KEGG pathways, over-represented by genes, induced by Abisil in normal fibroblasts Differentially expressed genes, cell pathways and processes at the organism level, wich are statistically significant over-represented in the GSEA-analysis, are presented.

It should be noted, that among the most represented molecular pathways induced by Abisil treatment in normal fibroblasts, a significant portion is related to longevity, including MAPK-, FOXO- and HIF-1 signaling pathways (Figure 1) [57].

It is noteworthy that in normal fibroblasts of the 13^th^ passage Abisil alters the expression level of a much larger number of genes than in the cells of the 6^th^ passage. When the selected threshold of expression was 2-fold or more (FDR <0.05) in the cells of the 6^th^ passage, Abisil activated 21 genes and repressed 16 genes, whereas in cells of the 13^th^ passage, the expression level of 43 and 67 genes were affected, respectively. This result may reflect the greater randomness of the expression response in old cells compared to younger ones. Some authors have mentioned the age-dependent increase of cell-to-cell variation in gene expression, so called increased transcriptional noise [58, 59]. Among the 31 upregulated genes which alter their activity only in fibroblasts of the 13^th^ passage, the most represented genes are of apoptosis (12 genes) and MAPK signaling pathway (5 genes). Among the 62 downregulated genes in fibroblasts of the 13^th^ passage, the *BTG2* gene is the one most associated with cellular aging in our opinion. Its expression has been shown to be important in negatively regulating cell proliferation [60]. Thus, terpenes of fir extract induce in normal fibroblasts genes of stress response, apoptosis regulation, and tissue regeneration.

In this study, fibroblast passaging was considered as one of the models of aging. Fibroblasts of the 13^th^ passage exhibit various external signs of cellular aging, such as inhibition of proliferation as compared with the 6^th^ passage (control). When comparing the expression of old and young fibroblasts 5804 differentially expressed genes were revealed generally.

The following aging-associated features of the expression profiles in fibroblasts should be noted: reduced expression of various cell adhesion molecules, chemokines expression, cyclin-dependent kinases, lamin, GADD45 family members, cAMP-dependent transcription factors (CREB), and PI3K/Akt overexpression. GSEA analysis allowed us to identify a number of biological processes, with the strongest changes in gene expression being (as part of this aging model): the suppression of angiogenesis, cell differentiation, MAPK cascade, chemotaxis, response to hypoxia, tissue regeneration and other processes, as well as modulation of intercellular communication, adhesion, migration, and ion exchange.

The potential geroprotector may be able to restore the expression level of genes in old cells, and transform them into younger cell gene expression profiles [61]. The possible mechanisms of the potential geroprotective effect of Abisil are:

- GADD45 (growth arrest and DNA damage inducible protein) gene family is associated with both tumor suppression and with the longevity. The expression level of GADD45 was reduced by 2-fold in fibroblasts of the 13^th^ passage compared with the 6^th^ passage. At the same time, Abisil treatment caused a 2-fold increase of expression level of GADD45B/G genes and 1.5-fold increase – of GADD45A.
- Abisil treatment is accompanied by a 1.5 to3-fold increase in the expression of heat shock proteins genes *HSPA1B*, *HSPA1A*, *DNAJB9* (*Hsp40 B9*), *DNAJB4* (*Hsp40 B4*), *HSPH1*, *DNAJB1* (*Hsp40 B1*), *HSPA9*, and others.
- Modulation of the cell cycle, in particular the MAPK signaling pathway.
- Modulation of NF-κB signaling pathway.
- Modulation of Toll-like receptor signaling pathway.
- Modulation of TGF-beta signaling pathway.

Also worthy of attention is the induction of compensatory changes for the following genes:

- Expression of tumor-suppressive regulators of MAPK-signaling cascade *DUSP5*, *DUSP1*, and *DUSP6* (dual specificity phosphatases) decreases with fibroblasts aging (in the 13^th^ passage compared with the 6^th^). Abisil treatment restores it to a level above the previous level.
- The expression level of *MYC* (v-myc avian myelocytomatosis viral oncogene homolog), *JUN* (jun proto-oncogene), *FOSB* (FBJ murine osteosarcoma viral oncogene homolog B), *FOSL1* (FOS-like antigen 1) protooncogenes decreases during fibroblasts aging. Abisil treatment restores it to a level of younger cells.
- The expression level of *SOCS3* (suppressor of cytokine signaling 3) decreases with aging. Abisil treatment restores it to a level of early passages.
- *CREB5* (cAMP responsive element binding protein 5) gene expression is decreased during aging and restored to the previous level after Abisil supplementation.
- *DDIT3* (DNA damage inducible transcript 3) gene expression is decreased during aging and restored to the previous level after Abisil supplementation.
- *KLF2* and *KLF4* (Kruppel-like factors 2 and 4) expression is decreased during aging and restored to the previous level after Abisil supplementation.
- *BMF* (Bcl2 modifying factor), *TRIB3* (tribbles pseudokinase 3), *BHLHE40* (basic helix-loop-helix family member e40), *TLR4* (toll-like receptor 4), *RGS4* (regulator of G-protein signaling 4), *GDF15* (growth differentiation factor 15), *NGFR* (nerve growth factor receptor), and *CTGF* (connective tissue growth factor) gene expression is increased during aging and restored to the previous level after Abisil supplementation.

Among global mortality rates for cancer, pancreas cancer takes 6^th^ place, and colon cancer takes 3^th^ place [62, 63]. We have studied Abisil's effects on the gene expression level in human cell lines of colon adenocarcinoma (*Caco-2*) and pancreas adenocarcinoma (*AsPC-1*).

It is noteworthy the increased expression level of all three genes (*GADD45A*, *GADD45B*, and *GADD45G)* related to the GADD45 family - both in normal fibroblasts and tumor cell lines (*AsPC-1* and *Caco-2)*. GADD45 family proteins are stress sensors and involved in the intersection of several cell signaling pathways, including apoptosis, DNA repair, and cell cycle arrest [64]. Defects in the *GADD45* genes often accompany the initiation and progression of malignancies, and GADD45 mediates the effects of multiple chemotherapeutic drugs [65]. For example, it has been shown that the sensitivity of prostate adenocarcinoma cell lines to docetaxel increased by enhancing the expression of *GADD45A*, but lack of *GADD45* expression, however, can cause inefficiencies of chemotherapy [66]. Simultaneously *GADD45* activity can have anti-aging effects as well [67, 68].

The overexpression of *DUSP1-2, DUSP4-6,* and *DUSP8* genes also should be noted. Many genes of the DUSP family are responsible for the suppression of MAPK signal transduction cascade, thereby being the tumor suppressor genes, that are responsible for the effectiveness of chemotherapy [69–74].

In both *Caco-2* and *AsPC-1* cancer cell lines Gene Ontology processes were enriched by overexpressed genes of apoptosis, intercellular signal transduction, and cellular response to organic substance (Table 1).

**Table 1:** The results of GSEA (GO) for the top 300 of overexpressed genes (*Caco-2* and *AsPC-1* cell lines, averaged)

GeneOntology process	Genes
Apoptotic process	*EGR3, INHBA, PDK4, JUN, CTGF, DDIT4, GDF15, EGR2, EPHA2, EGR4, CLCF1, MYC, GCLM, CXCR3, PPP1R15A, VEGFA, RFFL, OSGIN1, EGR1, GADD45B, CYR61, SNAI2, SOCS3, THBS1, FOSL1, SERPINE1, ID1, CEBPB, DDIT3, DUSP6, DSG3, EDAR, BMP2, PLK2, PLK3, ATF3, C8orf4, SPRY2, ANXA1, TNFRSF1A, PMAIP1, TNFSF15, ASNS, SOX9, NUAK2, RNF41, PIM1, CTH, SIAH2, TNFRSF10B, GATA6, SQSTM1, IER3, CHAC1, STK40, TCF7L2, GADD45A, SOCS2, RIPK2, BIRC3, RHOB, FOXO3, F3, TNFSF9, PAWR, WNT7B, ARHGEF2, ADM, H1F0, ZC3H12A, HERPUD1, HK2, NCF2, DUSP1, ANKRD1, AEN, BBC3, PLEKHG2, EDN1, CITED2, GADD45G*
Intercellular signal transduction	*INHBA, CXCL8, ARL14, JUN, CTGF, DDIT4, GDF15, EPHA2, CLCF1, MYC, CXCR3, PPP1R15A, VEGFA, RFFL, RND3, GADD45B, CCL20, DUSP4, CYR61, SNAI2, SOCS3, THBS1, EDN2, ID1, CEBPB, NFATC1, DDIT3, NFKB2, DUSP6, EREG, EDAR, BMP2, ARHGAP32, PLK2, FAM110C, PLK3, DGKD, ATF3, C8orf4, SPRY2, TNFRSF1A, TRIB1, PMAIP1, PLCH1, DUSP8, TNFSF15, SOX9, NUAK2, RNF41, CTH, SIAH2, TNFRSF10B, GAB2, SQSTM1, SESN2, IER3, LIF, CHAC1, CYTH1, SPSB1, DVL1, STK40, TCF7L2, GADD45A, SOCS2, RIPK2, BIRC3, CCDC88C, NFATC2, HRH1, SPRY4, RHOB, FOXO3, F3, CSF1, WNT7B, ARHGEF2, ARFRP1, ADM, FOS, HERPUD1, NCF2, DUSP1, ANKRD1, AEN, BBC3, DGKE, RAPGEF5, HBEGF, RAP1GAP2, PLEKHG2, WWC1, EDN1, GPAT3, AVPI1, GADD45G*
Cellular response to organic substance	*EGR3, INHBA, CXCL8, PDK4, JUN, CTGF, DDIT4, GDF15, EGR2, EGR4, CLCF1, MYC, CXCR3, PPP1R15A, VEGFA, RFFL, EGR1, CXCL3, CCL20, DUSP4, CYR61, SNAI2, SOCS3, THBS1, TGIF1, SERPINE1, EDN2, ID1, KLF6, CEBPB, DDIT3, NFKB2, DUSP6, KLF2, EREG, EDAR, BMP2, MT1G, ATF3, CPEB2, SPRY2, ANXA1, TNFRSF1A, TRIB1, TNFSF15, ASNS, SOX9, PIM1, CTH, GATA6, SQSTM1, SYBU, MT1X, OASL, SOCS2, RIPK2, NEDD4L, BIRC3, HRH1, DKK1, CALB1, FAM83G, FOXO3, MT2A, F3, CSF1, NR1D1, PAWR, WNT7B, ARHGEF2, FOS, ZC3H12A, HERPUD1, NCF2, DUSP1, HDAC5, ANKRD1, BBC3, JUND, HBEGF, PLEKHG2, EDN1, CITED2*

**Figure 2:**
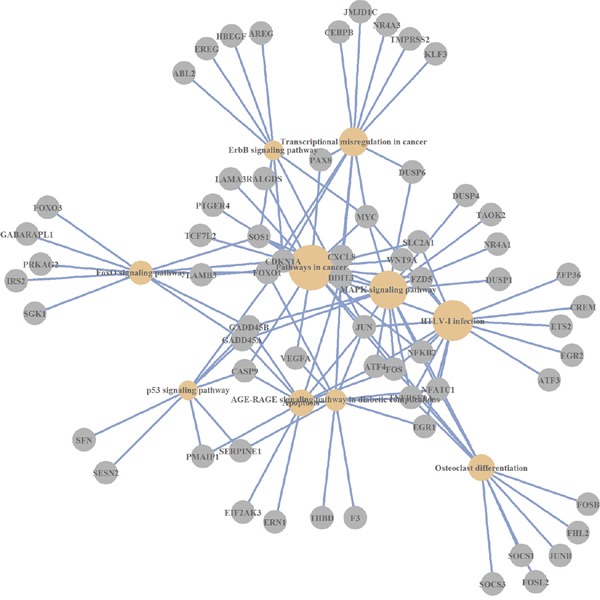
KEGG pathways, which are over-represented by differentially expressed genes (*AsPC-1* cell line)

**Figure 3:**
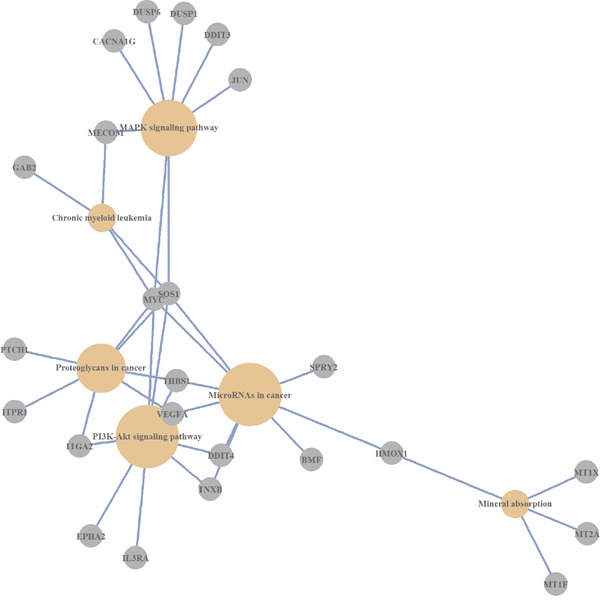
KEGG pathways, which are over-represented by differentially expressed genes (*Caco-2* cell line)

Thus, the analysis of over-represented pathways across differentially expressed genes revealed the number of cellular mechanisms that are modulated by Abisil (Figures 1–3). Among them: “MAPK signaling pathway”, “p53 signaling pathway”, “Apoptosis”, “Cell cycle”, “Transcriptional misregulation in cancer”, “HIF-1 signaling pathway”, “FOXO signaling pathway”, “TGF signaling pathway”, “TNF signaling pathway”, “Amino sugar and nucleotide sugar metabolism”, and “Protein processing in endoplasmic reticulum”. There are also a series of processes at the organismal level: “Longevity regulating pathway”, “Type II diabetes mellitus”, “Insulin resistance”, and “Infectious diseases (Influenza A, Legionellosis)”. The last one suggests the potential effect (it is difficult discuss about the direction of this effect a priori) ofAbisil on the penetration and proliferation of infectious agents or immune response.

As a result of the drug supplementation in all examined cell lines the expression level of several thousand genes changed. In general, the effects of increase in the expression level prevailed.

The top list includes both overexpressed onco-suppressors (gene of the GADD45, DUSP, and DDIT families), and proto-oncogenes (genes of c-Myc, c-Jun, EGR families and others.). Data indicate that Abisil exposure was associated with the modulation of key signaling pathways responsible for cell cycle control, proliferation, differentiation, apoptosis (e.g., MAPK, TNF, p53, FOXO, and TGF signaling pathways), cell-cell signaling, stress response, cAMP-dependent signaling and protein refolding.

Of particular note is the increase in the expression of all three members of the *GADD45* family genes which may serve as tumor suppressors. Chemotherapeutic drugs induced up-regulation of these genes is one of the factors, that determines the effectiveness of chemotherapy. Another important effect is the overexpression of most of the DUSP family genes responsible for the inhibition of the MAPK cascade, which plays a role in the response to chemotherapy as well.

The results of GSEA-Gene Ontology analysis allows one to make the assumption that the biological processes responsible for the negative regulation of apoptosis, prevail over the processes of induction of programmed cell death in normal human fibroblasts. In the cell lines *Caco-2* and *AsPC-1* the situation is reversed.

The potential geroprotector properties of Abisil may be conditioned by induced overexpression of both *GADD45* gene family, and the family of heat shock proteins *HSPA1A/A1B/A9*, *Hsp40 B1/B4/B9*, and *HSPH1*.

## MATERIALS AND METHODS

### Abisil composition

Pharmaceutical composition Abisil^®^ is a complex of terpenoids obtained from capsule extract *Abies sibirica* of Penaceae family enriched with monoterpenoids. Chemical and quantitative composition of the pharmaceutical composition of Abisil was studied with gas-liquid chromatography (GLC) by LCM-7A chromatograph (Chromatograph, Russia) using a thermal conductivity detector (TCD), packed column (length of 3.0 m, a diameter of 5 mm), and stationary phase “Apiezon L” (M&I Materials Limited, United Kingdom) on polychrome. The consumption of carrier gas (helium) was 30 ml/min, with a column temperature of 125°C, and a vaporization chamber temperature of 180°C. Identification of the main components was carried on the relative retention times and “bystander” compounds. The revealed Abisil composition is a standardized terpenoid substance derived from capsule extract of *Abies sibirica* (Table 2).

**Table 2:** The composition of Abisil

Component	Mass fraction	
	ppm	%
**Cyclic monoterpenes:**	**396969.3**	**16.9**
3-Carene	150186.4	6.4
Bicyclo[2,2,1]hept-2-ene, 2,3-dimethyl	2099.9	0.1
Cyclohexane, 4-methylene-1-(1-methyl ethyl)-	16793.4	0.7
Bicyclo[3,1,0]hex-2-ene, 2-methyl-5-(1-methylethyl)-	150186.4	6.4
Camphne	77703.2	3.3
**Acyclic monoterpenes:**	**248366.8**	**10.6**
Ocimene	52542.8	2.2
Santolina triene	195824	8.3
**Esters and Triterpene acids:**	**533050**	**22.7**
Phosphoric acid, tribornyl ester	380760.6	16.2
o-trifluoroacetyl-isopulegol	136912.8	5.8
Methyl abietate	15376.6	0.7
**Sesquiterpenes:**	**117968**	**5.0**
Longifolene-(V4)	61484.9	2.6
Longifolene-12	18411.6	0.8
Patchoulene	8553.3	0.4
gamma-Elemene	6164.8	0.3
Tricyclo[5,4,0,0(2,8) unden-9-ene, 2,6,6,9-tetramethyl	7912.0	0.3
beta-Humulene	14601.0	0.6
Dihydro-(-)-Neoclovene-(11)	840.4	0.0
**Sesquiterpenols:**	**104287.7**	**4.4**
Agarospirol	28098.4	1.2
Cubenol	2428.0	0.1
tau-Cadinol	2664.4	0.1
cis-Lanceol	1613.7	0.1
Humulane-1,6-dien-3-ol	2369.1	0.1
alpha-Bisabolol	67114.1	2.9
**Diterpenes:**	**18014**	**0.7**
Kaurene	1439.9	0.1
Trachylobane	3486.9	0.1
1H-Naphtho[2,1-b]pyran,3-e	13087.2	0.6
**Diterpenols:**	**16651.7**	**0.7**
Thunbergol	16651.7	0.7
**Aromatic hydrocarbons:**	**2988.1**	**0.1**
9,9'-Biphenanthrene	2988.1	0.1
**Steroids and Hormones:**	**46179.6**	**2.0**
9,9'-Biphenanthrene	1228.1	0.1
Resibufogenin	29023.1	1.2
9(11)-Dehydrotestosterone	15928.4	0.7
**Spirits:**	**1149.1**	**0.005**
1-Heptatriacotanol	1149.1	0.005
**Cycloalkanes:**	**541610.7**	**23.0**
1,3,5,6-Tetramethyladamantane	541610.7	23.0
**Diterpene acids:**	**323609.2**	**15.4**
Palustric acid	57733.0	2.5
Abietic acid	247651.0	10,5
beta-Pimaric acid	18225.2	2.5

The terpenoid composition is a thick liquid from yellow transparent to milky white in color and has a specific odor. It has certain physical constants, namely: an acid number (70-90 mg), a saponification number (100-130 mg), an ester number (10-60 mg), and an index of refraction (1,500 to 1,520).

### Cell culturing

Human pancreas adenocarcinoma cell line AsPC-1 (ATCC - CRL-1682) and colorectal adenocarcinoma cell line *Caco-2* (ATCC - HTB-37) were kindly provided by Dr. Peter Chumakov (EIMB RAS, Moscow, Russia). Cells were maintained in Dulbecco's modified Eagle's medium (DMEM, Invitrogen, USA) supplemented with 10% and 20% fetal bovine serum (Harlan Sera-Lab, Loughborough, UK) accordingly, 100 U penicillin per ml and 100 mkg streptomycin per ml (Gibco, Thermo Fisher Scientific, USA). Cells were cultured at 37°C in a 5% CO_2_ atmosphere and passaged every 2-3 days by dissociation with trypsin (Gibco, Thermo Fisher Scientific, USA).

Primary fibroblasts were provided by The Laboratory of Cell Cultures of the Institute of Medical Cell Technologies (Ekaterinburg, Russia). Cells were maintained in the medium described above (with 10% FBS). Cells were passaged, when the culture had reached approximately 80% confluence. In order to save unique properties of the model, cells were frozen in DMEM with 7% DMSO and 30% FBS after the 4^th^ and 10^th^ passages. Effects were studied on the 6^th^ and 13^th^ passages.

Cells in 70% confluence were treated for 6 hours with dilution of Abisil (1.2 mg/ml) in DMEM with 2% FBS. After that, the culture medium was replaced with a fresh medium and in 18 hours cell viability was analyzed using MTS test (Promega, USA).

All cells including control cell lines were plated in triplicate. RNA extraction for further analysis was performed right after treatment with active substance dilution.

### RNA extraction and quality control

Total RNA was extracted from 24 samples using RNeasy Mini kit (Qiagen, Germany), including 4 cell lines after treatment (*Caco-2*, *AsPC-1*, and primary fibroblasts at the 6^th^ and 13^th^ passages) and corresponding control cells (all in triplicates). RNA quality and quantity was determined with the Agilent 2100 Bioanalyzer (Agilent Technologies, USA) and the Qubit 2.0 Fluorimeter (Thermo Fisher Scientific, Invitrogen, USA), respectively. RNA samples with an RNA integrity number (RIN) higher than 8.0 were used for downstream analysis.

### RNA library preparation and sequencing

Total RNA (2 μg) from each sample was used for mRNA library preparation with a TruSeq RNA Sample Preparation Kit v2 Low Sample (LS) protocol (Illumina, USA) according to the manufacturer's instructions. The quality and concentration of cDNA library was assessed using an Agilent 2100 Bioanalyzer (Agilent Technologies) and a Qubit 2.0 fluorometer (Invitrogen), respectively, before sequencing. To optimizing cluster densities, the libraries were quantified by qPCR. cDNA libraries were sequenced (single end reads, 75 bp) on an Illumina NextSeq 500 platform (EIMB RAS “Genome” center, Russia).

### Processing of RNA-Seq data and differential expression analysis

The raw single end reads were quality controlled and trimmed using FastQC (http://www.bioinformatics.babraham.ac.uk/projects/fastqc/) and Trimmomatic (http://www.usadellab.org/cms/index.php?page=trimmomatic) tools with default parameters. The clean reads were separately aligned to the human reference genome (GRCh38) in the Ensembl (release 80) using TopHat2 software (http://ccb.jhu.edu/software/tophat/index.shtml). The read counting was performed with HTSeq-count (http://www-huber.embl.de/HTSeq/doc/overview.html). The differentially expressed genes were determined with the p-value ≤ 0.01 using the edgeR, lme4, biomaRt, Rgraphviz, topGO, ggplot2, pathview, and clusterProfiler analysis packages in R.

## SUPPLEMENTARY TABLE




