# Supplementary material for: Effects of Abies sibirica terpenes on cancer- and aging-associated pathways in human cells
Source: Oncotarget. 2016 Nov 19;7(50):83744–54. doi: 10.18632/oncotarget.13467 (PMC5347801; doi:10.18632/oncotarget.13467)
Supplement: Supplementary file 1 [file oncotarget-07-83744-s001.pdf]

Effects of *Abies sibirica* terpenes on cancer- and aging-associated pathways in human cells

SUPPLEMENTARY TABLE

Supplementary Table S1: Defferential expression induced by Abisil in different cell lines

See Supplementary File S1.
